# An Autonomy-Supportive Online Decision Aid to Assist Smokers in Choosing Evidence-Based Cessation Assistance: Development Process and Protocol of a Randomized Controlled Trial

**DOI:** 10.2196/21772

**Published:** 2020-12-15

**Authors:** Thomas Gültzow, Eline Suzanne Smit, Raesita Hudales, Vera Knapen, Jany Rademakers, Carmen D Dirksen, Ciska Hoving

**Affiliations:** 1 Department of Health Promotion Care and Public Health Research Institute Maastricht University Maastricht Netherlands; 2 Department of Communication Science Amsterdam School of Communication Research University of Amsterdam Amsterdam Netherlands; 3 Netherlands Institute for Health Services Research (Nivel) Utrecht Netherlands; 4 Department of Family Medicine Care and Public Health Research Institute Maastricht University Maastricht Netherlands; 5 Department of Clinical Epidemiology and Medical Technology Assessment Care and Public Health Research Institute Maastricht University Medical Centre Maastricht Netherlands

**Keywords:** digital health, decision making, decision support technique, decision aids, smoking, smoking cessation, informed decision making

## Abstract

**Background:**

Decision aids (DAs) may be used to facilitate an autonomous, informed decision to cease smoking and promote the uptake of evidence-based cessation assistance (ie, behavioral support, nicotine replacement therapy, or prescription medication). However, knowledge is lacking regarding their effective elements and (cost-)effectiveness.

**Objective:**

We describe the development process of an online DA (called “VISOR”) that helps smokers to choose evidence-based cessation assistance. Additionally, we provide a description of the protocol of an ongoing randomized controlled trial in which the DA containing an explicit value clarification method (VCM) and tailored advice is compared with a DA without an explicit VCM and tailored advice.

**Methods:**

The development of “VISOR” was based on the International Patient Decision Aid Standards guidelines. Viewpoints of end users (collected through 20 interviews with smokers) and clinical and scientific experts (assessed using 2 Delphi studies with 24 scientists and 38 clinicians) were assessed regarding cessation tool decision making and preferred DA content. These findings, together with principles from the Self-Determination Theory, served as input for the development of the online DA. A first DA prototype was alpha-tested in September 2019 and beta-tested for usability in December 2019; feedback was incorporated and resulted in a final version. The final DA contains (1) an information section, (2) an optional knowledge quiz, (3) a brief smoking assessment, (4) intuitive decision, (5) intermediate advice, (6) an explicit VCM, (7) tailored advice, and (8) access information. A randomized controlled trial is currently being conducted to assess the DA’s (cost-)effectiveness compared to a DA that does not include the explicit VCM and the tailored advice; specifically, the DA’s effect on smoking abstinence, uptake of evidence-based cessation assistance, smoking abstinence mediated through uptake of evidence-based cessation assistance, and decisional conflict are investigated. Participants are randomly allocated to receive access to 1 of the 2 DAs and are asked to complete 5 questionnaires (including the baseline questionnaire) over a period of 12 months. To evaluate the effects of the DA on the outcome measures, logistic and linear regression analyses as well as mediation analyses will be carried out. An economic evaluation will be performed to assess the cost-effectiveness.

**Results:**

Data regarding the effect of the VISOR DA are currently being collected, and data collection is expected to be concluded in 2021.

**Conclusions:**

By making use of an iterative process that integrated different stakeholders’ perspectives (including end users), we were able to systematically design an evidence-based DA. The study will contribute to the current knowledge regarding smoking cessation DA application, the added value of explicit VCMs, and the effect of behavioral and informed decision-making outcomes.

**Trial Registration:**

Netherlands Trial Register NL8270; https://www.trialregister.nl/trial/8270

**International Registered Report Identifier (IRRID):**

DERR1-10.2196/21772

## Introduction

Smoking continues to kill, both on a global level [1] and in the Netherlands [2]. According to predictions, more than 8 million deaths per year will be caused worldwide by tobacco use by 2030 if evidence-based smoking cessation interventions are not put into use [3]. One way to reduce tobacco-related deaths is to promote the uptake of evidence-based cessation assistance, such as pharmacological support (eg, nicotine replacement therapy [NRT]) [4] and behavioral support (eg, counselling) [5]. Unlike non-evidence–based cessation assistance, such as acupuncture [6], evidence-based cessation assistance can greatly increase successful smoking cessation [7]. However, uptake of evidence-based cessation assistance is low in the Netherlands [8]. And, even if people are interested in using evidence-based cessation assistance, they still have to choose between the multitude of options that exist in order to make an informed decision. Making an informed decision requires people to gather and review information regarding cessation assistance options and weigh up all the advantages and disadvantages of those options [9]—tasks that can be difficult for unsupported lay people [10].

Decision aids (DAs) are interventions that are specifically designed to help users with those difficult decisional processes. DAs aim to facilitate informed decision making between different health or health care options by providing information and helping users to become aware of their own values ​​in relation to those options [9]. Often, DAs are used when people have to choose between different medical treatment options or if they are considering whether to participate in a screening program [9]. However, a systematic review regarding DAs for smoking cessation has also shown positive results in terms of increased quit attempts [11]. Insights from the Self-Determination Theory (SDT) also suggest that the offering of choice (as DAs explicitly do) enables individuals to engage in long-term behavior change, such as smoking cessation, by supporting their need for autonomy [12,13]. Nonetheless, only one DA for smoking cessation has previously been developed and tested in the Dutch setting [14]. And, while this DA was effective in promoting quit attempts and smoking abstinence, no effects were found regarding the use of cessation assistance [14]. As increased cessation assistance uptake could further improve cessation outcomes [7], we propose several limitations of the earlier DA that might need to be overcome to increase cessation assistance uptake and subsequent smoking cessation behavior. First, the earlier DA was paper-based and sent to people by mail, thereby limiting widespread dissemination. Offering a smoking cessation DA online could potentially reach many more people [15], especially given that the Dutch are likely to search for health-related information online nowadays [16]. Second, offering the DA online allows for a more flexible and interactive design that can be particularly interesting for people with a high need for autonomy [17]. And third, the aforementioned DA did not explicitly include methods that help users to become aware of their own values, even though explicitly including smokers’ values in their decisional process could potentially improve cessation assistance uptake rates [18,19].

So-called value clarification methods (VCMs, also referred to as value clarification exercises) can support users to “evaluate the desirability of options or attributes of options within a specific decision context, in order to identify which option he/she prefers” as defined by the International Patient Decision Aid Standards (IPDAS) [20]. The underlying belief is that users with clarified values (ie, users who know what is important to them) will be more likely to choose an option that reflects their own preferences, which is regarded as a prerequisite of high-quality decision making [9,20]. VCMs can either be explicit or implicit; the former refers to methods that involve the user actively engaging in an activity (eg, scoring certain statements), while the latter refers to the provision of information that is specifically linked to the decision at hand (similar to the aforementioned paper-based DA [14]), with the underlying belief that users will engage in cognitive processes themselves to reach a decision [21]. There is some evidence that shows that explicit VCMs are more effective than implicit VCMs (in terms of decisional processes) [22], especially in the long run [21] and when people are supported in understanding the implications of their clarified values for the decision [23,24]. One way to help users understand these implications is to show participants which options fit their clarified values [23] (ie, tailored advice following the VCMs based on the answers that were provided). Interestingly, the feasibility study of a digital DA for smoking cessation that did include an explicit VCM has shown promising results, both in terms of evidence-based cessation assistance uptake and smoking cessation outcomes [25]. Thus, explicit VCMs could potentially be used to enhance the effectiveness of DAs for smoking cessation. However, the feasibility study’s design (pretest and posttest assessment without a control group) limits the interpretability of the feasibility study’s findings [25], and it remains unclear whether such an explicit VCM results in significant changes in cessation rates and decisional outcomes.

Based on these limitations and the existing literature, an online DA, called “VISOR,” containing an explicit evidence-based VCM and tailored advice was developed that helps smokers to choose evidence-based cessation assistance tools. This DA was systematically developed and is currently being tested in a national randomized controlled trial (RCT) for its effect on individual decisional processes and smoking cessation attempts, as well as smoking abstinence compared to a DA without the explicit VCM component. Specifically, we aim to test the following hypotheses. 

H_1a/b/c_ is that a DA with an explicit VCM and tailored advice will lead to a statistically significant increase in smoking abstinence after 1 month (H_1a_), 6 months (H_1b_), and 12 months (H_1c_) compared to a DA without an explicit VCM and tailored advice: direct effect on smoking abstinence.

H_2a/b/c_ is that a DA with an explicit VCM and tailored advice will lead to a statistically significant increase in evidence-based cessation assistance use after 1 month (H_2a_), 6 months (H_2b_), and 12 months (H_2c_) compared to a DA without an explicit VCM and tailored advice.

H_3a/b_ is that the positive effect of a DA with an explicit VCM and tailored advice (vs a DA without an explicit VCM and tailored advice) on smoking abstinence after 6 months and 12 months will be at least partially mediated by the use of evidence-based cessation assistance at 1-month (H_3a_) and 6-month (H_3b_) follow-ups, respectively: indirect effect on smoking abstinence.

H_4_ is that a DA with an explicit VCM and tailored advice will lead to a statistically significant decrease in decisional conflict (state of uncertainty about which course of action to take) right after the DA compared to a DA without an explicit VCM and tailored advice.

In addition, it will also be tested whether the DA is more cost-effective compared to a DA without the explicit VCM component. This paper aims to thoroughly describe the DA “VISOR,” its development process, and the study protocol to test its (cost-)effectiveness.

## Methods

An RCT is being conducted and will be reported in line with the CONSORT-EHEALTH checklist [26]. Participants are randomly allocated to receive access to either a DA with an explicit VCM and tailored advice (intervention group) or a DA without an explicit VCM and tailored advice (control group). Study materials (such as questionnaires, but not the DA) will be made available on the open science framework website, in line with recommendations in the field of behavioral science [27].

The study does not fall under the scope of the Medical Research Involving Human Subjects Act as indicated by the Medical Ethics Committee Zuyderland, the Netherlands (16-N-227). The development of “VISOR” and the accompanying studies are funded by the Dutch Cancer Society, UM2015-7744.

### Study Population

Recruitment is predominantly conducted online to reflect the online nature of the DA. We make use of various methods: Mainly, we are recruiting participants through project social media accounts (eg, [28]) and paid social media advertisements. We decided to make use of 4 big social media platforms (ie, Facebook, Instagram, Twitter, and LinkedIn) as they cater to different target groups [29]. In addition, our recruitment messages are being shared on the social media accounts of the project members and their institutions. Also, we promote the RCT through the project’s website, and we asked various relevant Dutch institutes and organizations (eg, the Dutch institute for addiction and mental health and municipal health services) to promote the study. Finally, we advertise the study in online newspapers. All recruitment materials include a link to enroll in the RCT, if possible, to simplify the process for potential participants as much as possible. Study inclusion criteria are: (1) participants are currently smoking, (2) participants are motivated to stop smoking within 6 months, (3) participants are 18–100 years old, (4) participants are able to understand Dutch, and (5) participants have access to the internet and have the necessary internet literacy skills to use the intervention. People are excluded if they only use e-cigarettes; however, dual users can participate. The rationale behind the inclusion and exclusion criteria is described in Table 1.

**Table 1:** Overview of the randomized controlled trial inclusion and exclusion criteria, including rationale.

Criteria			Rationale
**Inclusion criteria**			
	Participants are currently smoking	To test the primary hypothesis that the DA will have a positive effect on smoking cessation, it has to be tested among smokers	
	Participants are motivated to stop smoking within 6 months	As smokers unmotivated to quit cannot be expected to be willing to consider the use of cessation assistance	
	Participants are 18–100 years old	As the DA was developed for adults	
	Participants are able to understand Dutch	To ensure that participants understand all provided information	
	Participants have access to the internet and have the necessary internet literacy (skills) to use the intervention	As the DA is fully online, participants need both access to the internet and the necessary internet literacy (skills) to access and use the DA	
**Exclusion criteria**			
	Participants exclusively use e-cigarettes	As there is no consensus on whether cessation assistance tools can be used to cease e-cigarette use	

### Required Sample Size

The power calculation was based on the dichotomous outcome measure of smoking abstinence (primary outcome: 7-day point prevalence abstinence). The only previous RCT in the Netherlands testing the effect of a DA for smoking cessation on this outcome measure showed a significant effect (20.2% vs 13.6%) at 6 months [14]. To be able to significantly (α=.05; β=.20) detect the same effect in a 1-sided test, 398 smokers per arm are necessary at the end of the trial (796 in total). Considering 50% attrition over the intervention period, we aim to include 1592 smokers at baseline.

### The Intervention

#### Initial DA Development

The development process of the DA was based on the IPDAS guidelines for DA development [30]. In line with these guidelines, both end users’ (ie, smokers) and clinicians’ needs were assessed. In addition, we asked scientific smoking cessation experts for extra input as smoking cessation is often done without consulting clinicians — especially in the Netherlands [8]. In the end user needs assessment, 20 interviews were conducted to assess end users’ needs regarding potential cessation assistance’s characteristics that should be described in the DA and DA functions (eg, a knowledge quiz). The input of the experts was gathered through 2 Delphi studies (24 scientists and 38 clinicians completed the final round). The input of these 3 qualitative studies was used to inform the design and content of the DA, supplemented by the IPDAS background papers (eg, [31]) as well as other relevant literature in the field (eg, [23]) and in consultation with established experts in the field. The SDT served as the theoretical framework. After this, a first prototype was developed that was alpha-tested in September 2019 with potential end users (n=3) and Dutch smoking cessation experts (n=8). During the alpha test, participants were asked to focus on the content of the DA. The end users alpha-tested parts of the DA, while the smoking cessation experts tested the whole first version. Before the beta test, the DA was evaluated by 1 of the co-authors (JR) with regard to the comprehensibility of the text for people with limited health literacy. On the basis of that evaluation, some sections and sentences were rephrased or simplified.

#### Usability Testing

In December 2019, after the alpha test, the DA was beta-tested among 15 experts and end users: 5 smoking cessation counselors, 5 eHealth experts (of whom, 2 had digital DA experience), and 5 potential end users (ie, people motivated to stop smoking). Respondents were given access to the DA to assess the DA’s usability using the heuristic evaluation method (smoking cessation counselors and eHealth experts) and the think-aloud method (potential end users) [32].

Using the heuristic evaluation method, smoking cessation counselors and eHealth experts were asked to evaluate the DA against a list of recognized usability principles: (1) use simple and natural dialogue, (2) speak the user’s language, (3) minimize memory load, (4) be consistent, (5) provide feedback, (6) provide clearly marked exits, (7) provide shortcuts, (8) provide good error messages, (9) prevent errors, and (10) provide help and documentation. The smoking cessation counselors and eHealth experts were asked to use predetermined scenarios (eg, whether cessation attempts have been undertaken before) during the evaluation.

With the think-aloud method, end users were asked to complete the DA while verbalizing their thoughts. The think-aloud method is considered particularly useful in understanding processes of cognition and is considered to be of high value in evaluating an intervention’s design on usability flaws [32].

Data gathered through these tests were compared and compiled into a summary describing the usability flaws of the DA. Based on these results, adjustments to the DA were identified, which can be seen in Textbox 1.

**Overview of alterations to the decision aid (DA).**

- More information was added on how to use the DA: for example, “Now click on 'next' to start with the information.”
- The language and complexity level have been simplified.
- Certain subparts (eg, results of the knowledge quiz) were rewritten in a more positive tone.
- Content was shortened if possible (eg, by combining pages or by removing too many details).
- Some content was added (eg, required duration of prescription medication use).
- More visuals were added (eg, icon arrays to showcase cessation assistance’s effectiveness).
- Information regarding cessation assistance’s effectiveness was adapted to make it more accessible.
- The order of the given information was changed (eg, information regarding non-evidence–based cessation assistance was moved to the beginning as testers found it confusing that this information was mentioned only at the end of the information section).
- A function was added to enlarge visuals and tables.
- The layout was changed to make the DA more accessible to users with visual impairments (eg, we added more lines and changed the color of the text).
- All abbreviations were removed, as were references to terms that were used in other parts of the DA.
- All hyperlinks were removed.
- Two possible options (ie, combination of behavioral support and nicotine replacement therapy; combination of behavioral support and prescription medication) were added.
- A ranking of the evidence-based cessation assistance tools based on the value clarification method was added.

#### Description of the DA

An interactive Dutch DA with an explicit VCM and tailored advice that facilitates the process of choosing an evidence-based cessation assistance was developed. The DA follows a stepwise approach and is designed to encourage a decision that is autonomous and consistent with participant’s values and smoking behavior. The DA consists of 8 sequential sections: (1) information section, (2) optional knowledge quiz, (3) brief smoking assessment, (4) intuitive decision, (5) intermediate advice, (6) explicit VCM, (7) tailored advice, and (8) access information.

In step 1, the information section explains the decision at hand, as well as all the available cessation assistance in the Netherlands. The following topics are addressed: (1) smoking cessation with and without evidence-based cessation assistance, (2) non-evidence–based cessation assistance, and (3) evidence-based cessation assistance.

For the first topic, differences in smoking cessation outcomes are described between people using and not using evidence-based cessation assistance using simple frequency formats (eg, “By using one or more of these stop methods, 15 out of 100 people are still smoke-free after one year.”).

For the second topic, a list of various non-evidence–based cessation assistance tools (ie, acupuncture, laser therapy, hypnotherapy, mindfulness, smartphone apps, self-help books, and e-cigarettes) are described, and a remark is made that the use of these tools is not advised.

For the third topic, evidence-based cessation assistance is described (Table 2), including behavioral support in general, in which users can choose to read more detailed information about all possible options (ie, face-to-face counseling, counseling over the phone, group coaching, and eHealth); NRT in general, in which users can choose to read more detailed information about all possible options (ie, nicotine patches, nicotine gum, nicotine lozenge, nicotine mouth spray, and nicotine inhaler); prescription medication in general, in which users can choose to read more detailed information about all possible options (ie, varenicline, bupropion, and nortriptyline); and cessation assistance during pregnancy and breastfeeding. Users can compare all cessation assistance options using an option grid and are informed about combinations of multiple cessation assistance options. In addition, costs and reimbursement are described, and sources used to inform the information section content (ie, reports, scientific manuscripts, websites) are provided.

**Table 2:** Overview of cessation assistance characteristics included in the decision aid (DA).

Cessation assistance characteristic	Rationale for inclusion in the DA	This characteristic is included in the statements (VCM^a^) for:
Effectiveness of the cessation assistance options	During the interviews with potential end users, this was both the most mentioned characteristic and considered the most important characteristic.	Behavioral support, prescription medication
Probability of nausea and dizziness when the cessation assistance option is used	Probability of side effects was the third most often mentioned characteristic during the interviews with potential end users and was also considered to be of importance by the clinicians. The first 3 side effects were specifically named as important by potential end users, while the fourth was added, as this is a common side effect. Other side effects were included if they are regarded as being very likely to occur.	Nicotine replacement therapy, prescription medication
Probability of mood changes when the cessation assistance option is used		
Probability of headaches when the cessation assistance option is used		
Probability of sleeping problems when the cessation assistance option is used		
Probability of other side effects that are very likely to occur when the cessation assistance option is used		
Extent and type of contact someone has with a professional when the cessation assistance option is used	Both scientists and potential end users indicated that this was of great importance. Clinicians indicated that required contact with a health care professional was of great importance.	Behavioral support
How the cessation assistance option is used	This was integrated after it had been decided that Dutch health insurance companies would cover evidence-based cessation assistance use, as that left too few features to distinguish between the different nicotine replacement therapies and prescription medication.	Nicotine replacement therapy, prescription medication

^a^VCM: value clarification method.

In step 2, users can choose to complete the knowledge quiz or skip the knowledge quiz and proceed to the following step. If they complete the knowledge quiz, they will receive the results, see the correct answers to the questions that they answered incorrectly or could not answer (seeing the correct answers can also be skipped even if a respondent opts to do the knowledge quiz), and proceed to the following step.

The brief smoking assessment in step 3 consists of 2 closed (yes/no) questions: “To ensure that our advice suits you best, we would like to ask you if you smoke more than 10 cigarettes (or other tobacco products) on a normal day?” and “To ensure that our advice suits you best, we would like to ask you if you have ever made one or more smoking cessation attempts in the past that were unsuccessful?”

In step 4, an intuitive decision occurs between different clusters of cessation assistance tools: behavioral support; NRT; combination of behavioral support, NRT, and prescription medication; combination of behavioral support and NRT; combination of behavioral support and prescription medication; other, non-evidence–based cessation assistance; and no cessation assistance at all.

In step 5, intermediate advice is provided for users to reevaluate their intuitive decision in step 4 when they chose behavioral support only or NRT only, while also affirming at least 1 of the 2 questions in step 3 (indicating that they smoke more than 10 cigarettes on a normal day or have made one or more smoking cessation attempts in the past): They are advised to consider using a combination of behavioral and pharmacological cessation assistance tools. Intermediate advice is also given to users who chose non-evidence–based cessation assistance or no cessation assistance at all regardless of their answers in step 3: They are advised to consider using one or more evidence-based cessation assistance tools.

The explicit VCM in step 6 is provided for users that chose evidence-based cessation assistance tools in steps 4 or 5; users are asked to rate certain statements regarding cessation assistance characteristics (eg, “I prefer a stop method that works better, even if that means that I have to leave the house.”). These statements are also described in Table 2. Users only rate statements for options that belong to the cluster of cessation assistance options they selected in the previous step.

Step 7 is tailored advice based on the explicit VCM and an optional ranking of all options. The advice is given only when it is possible to give clear advice (ie, if users’ scores do not suggest that more than 2 cessation assistance tools are suitable based on their indicated values).

Step 8 involves accessing information on how to obtain the chosen cessation assistance (eg, nicotine patches).

**Figure 1:**
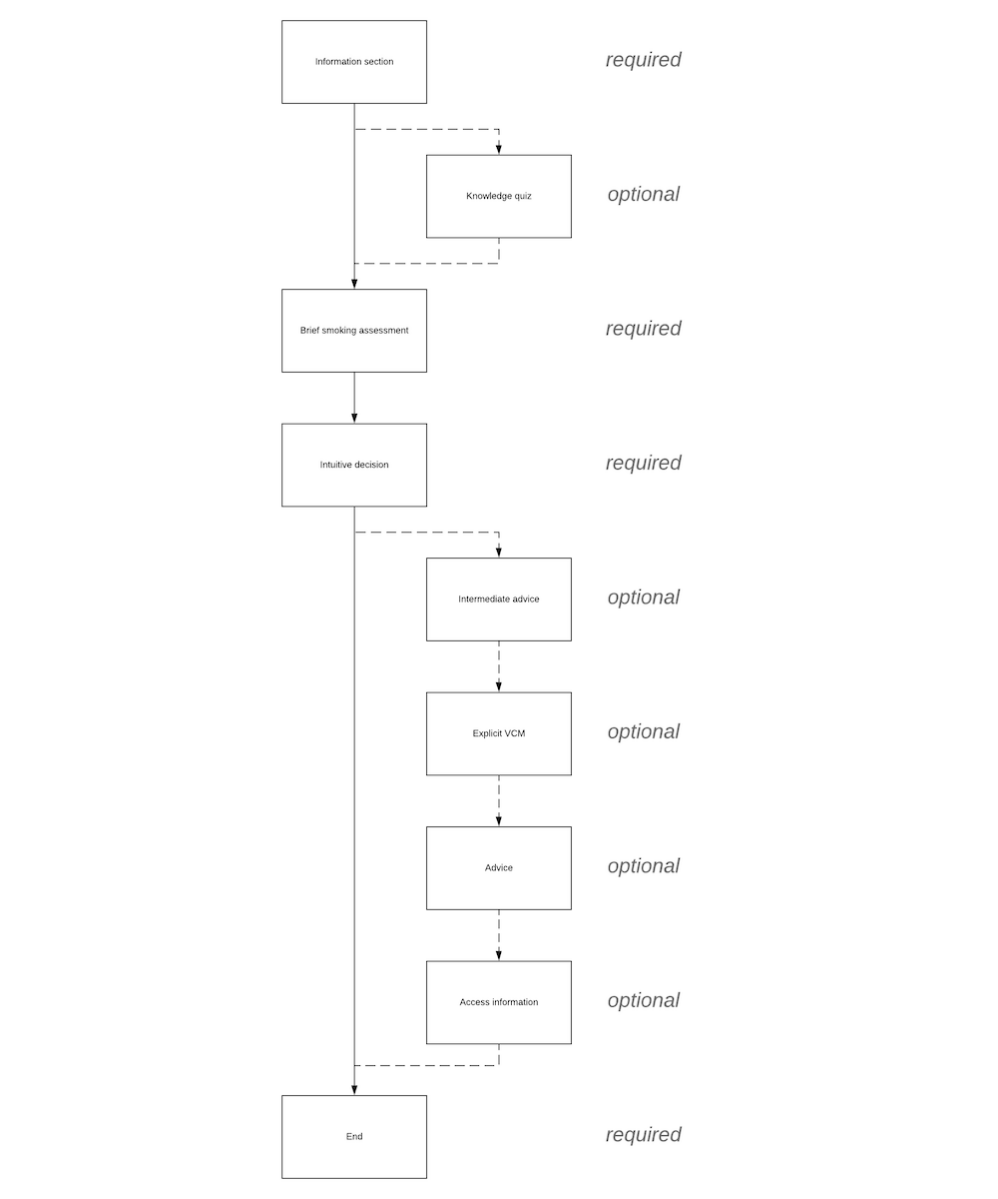
Sections of the decision aid. VCM: value clarification method.

**Figure 2:**
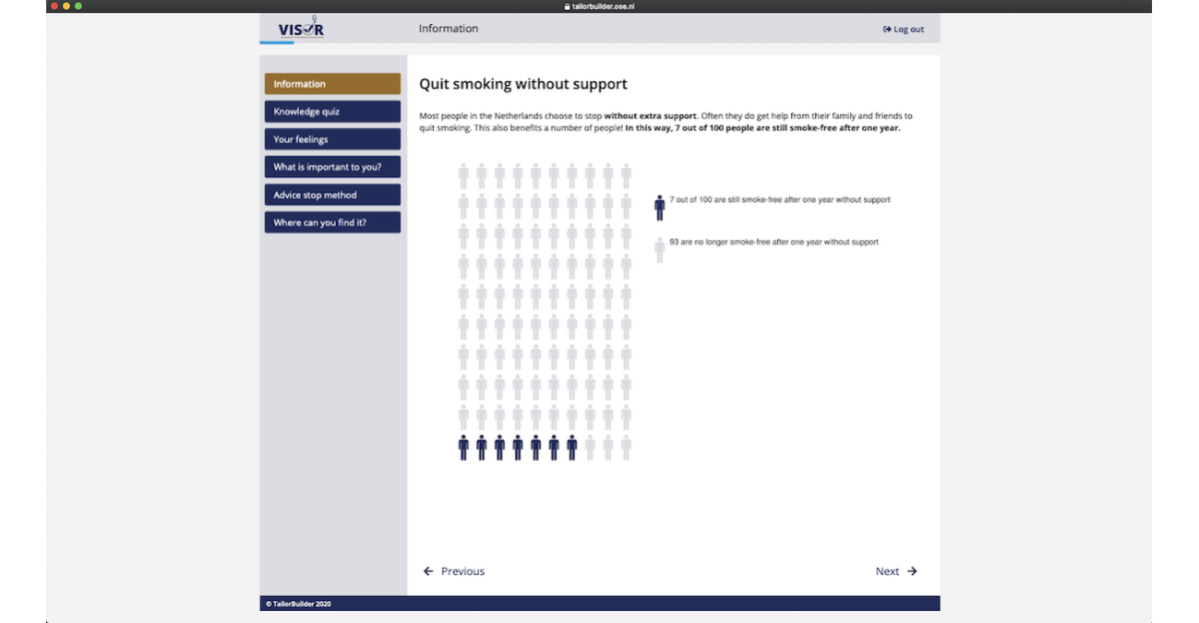
Screenshot of the information section in the decision aid (original text translated from Dutch).

**Figure 3:**
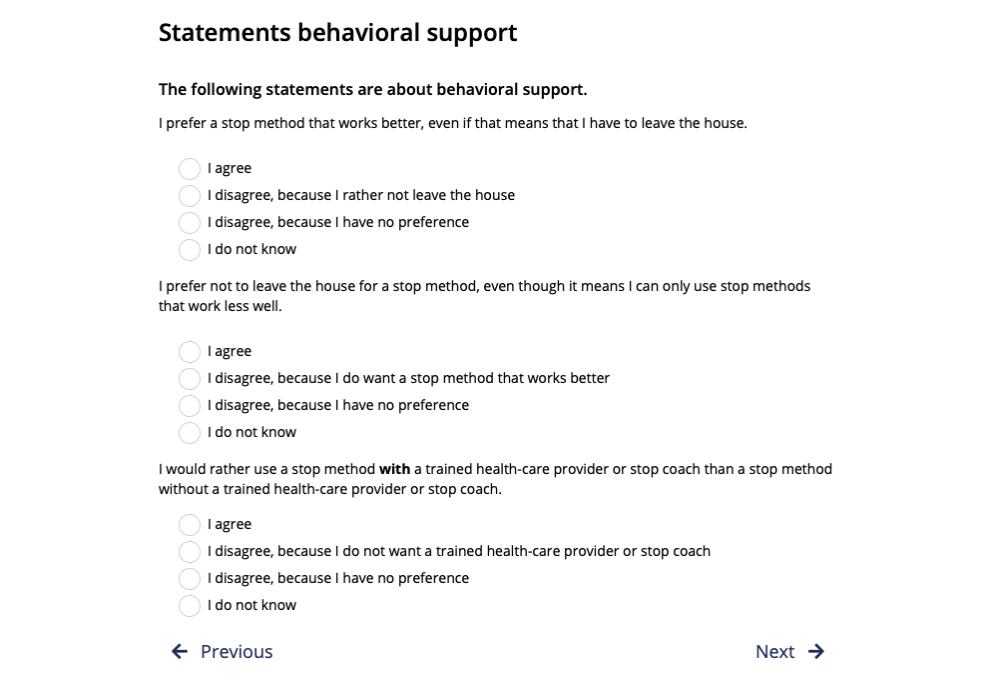
Screenshot of the value clarification method (VCM) in the decision aid (original text translated from Dutch).

Above and beyond these 8 steps, framing throughout the DA is positive and autonomy-supportive [33] (in line with the SDT as theoretical framework) to support users’ need for autonomy (eg, we refrained from using fear appeals [34] and controlling language [12,33]). We also clearly communicate that users can make their own choice at the end and included “cues” to support this process (again in order to support users’ need for autonomy; eg, by stating that the advice is only a recommendation and not a command and that it might be a good idea to discuss this with either the social environment, a health care provider, or both). A flow chart of the DA can be seen in Figure 1. The digital DA is hosted by the Dutch company OverNite Software Europe BV, and we make use of their product “TailorBuilder” [35]. Translated screenshots of different sections can be seen in Figure 2 (example of the information section) and Figure 3 (example of the explicit VCM).

#### Theoretical Background of the Different Sections

An information section was predominantly included as relevant knowledge is needed to engage in high-quality decision making [36] and as it is regarded as an active ingredient by the IPDAS [37,38]. Given this fact, and as potential end users indicated that they would like a knowledge quiz within the DA, a knowledge quiz was included at the end of the information section. Users are thus able to check for themselves if they have the necessary knowledge to engage in the decision process and can obtain additional information if desired. The content of the information section was mainly based on Dutch guidelines (eg, [39]), supplemented by various Cochrane reviews (eg, [40]), online information from the Dutch National Health Care Institute [41], and the Dutch Medicines Evaluation Board [42].

An intuitive decision to limit the choice set for the explicit VCM was included to both prevent choice overload [43] and to include intuitive decision-making processes in the DA. Choice overload describes a scenario in which the difficulty of the decision to be made is greater than the cognitive means of the person faced with the decision, due to too many options. Many DAs focus on a rather small set of available options [44] (eg, deciding between intensive or less intensive aftercare after breast cancer treatment [45]); yet, a cessation assistance DA has to include more options to present all available possibilities. Considering that the comparison of too many characteristics of too many options can lead to choice overload, which can in turn lead to worse decisional outcomes [43], in step 4 of the DA we decided to have users intuitively choose to go on with a limited choice set (eg, only behavioral support). Another benefit of this approach is that users are encouraged to make use of both deliberative and intuitive decision-making processes. While many DAs focus on facilitating deliberation, de Vries et al [36] have argued that combining those 2 strategies can lead to improved decisional outcomes. Importantly, however, users receive information about all possible options before they intuitively make a first, broad decision as knowledge is regarded as a necessary prerequisite for high-quality intuitive decision making [36].

As the Dutch smoking cessation guideline indicates that a combination of both behavioral and pharmacological support should be considered if smokers smoke >10 cigarettes per day or if many of their past cessation attempts failed [39], users that indicated in step 3 that this applies to them are provided with information on the improved effectiveness of a behavioral and pharmacological combination. Of course, even then, they can autonomously decide to continue with another cessation assistance set — in line with their preference and the SDT. In case smokers choose to use either no cessation assistance or no evidence-based cessation assistance, they are also reminded that evidence-based cessation assistance will probably lead to more successful cessation outcomes, but if they choose not to change their mind, they are directed to the last step (ie, access information) if they decided to use no cessation assistance or directly to the end if they decided to use no evidence-based cessation assistance.

We decided to use an explicit VCM in the intervention group as VCMs are regarded as active ingredients by the IPDAS [20,37], and while conflicting evidence exists [46], an overwhelming amount of research has shown that interactive methods to clarify people’s values seem to result in better decisional outcomes [22], especially in the long run [21] and if people are supported in understanding the implications of their clarified values [23,24]. This is why we decided to integrate individual tailored advice that is supplemented with information on how to obtain the different cessation assistance options to remove barriers for the users.

### Study Comparator Group

Participants in the control group receive the same DA without an explicit VCM and tailored advice; thus, the DA includes the following steps: (1) information section, (2) optional knowledge quiz, (3) brief smoking assessment, (4) intuitive decision, (5) intermediate advice, and (8) access information. Steps 6 and 7 are skipped. The only other difference is that participants in the control group are not immediately directed towards the end after they have chosen to use no evidence-based cessation assistance to also provide them with a chance to reevaluate their choice. More information about the individual steps can be found under “Description of the DA.”

### Trial Flow and Measurement Instruments

As participants register for the study via an online form, which includes their provision of informed consent and the creation of an account, participants are automatically randomized into 1 of the 2 groups (intervention or control group), allocating approximately 50% of respondents to either group. After this, they are asked to fill in the first part of the baseline questionnaire (t=0) consisting of 22-94 items (depending on the respective answers; eg, if the participants state that they have never attempted to cease smoking, no follow-up questions are posed), in which general demographic information (eg, age), smoking behavior, nicotine dependence, productivity loss, health care utilization, quality of life, and stages of decision making are assessed. Participants are excluded from the study if they indicate that they are <18 years old, do not smoke, are not motivated to stop smoking within 6 months, or only use e-cigarettes. Participants immediately receive access to 1 of the 2 DAs after being randomized, as this process is fully automated. After having accessed the DA, the second part of the baseline questionnaire is made available (t=1). The second part consists of 53-56 items regarding stages of decision making, the decision, the decision-making process, knowledge, perceived autonomy support, perceived competence, user evaluation, and recruitment channels.

One month after the baseline questionnaire (t=2), users are asked to fill in a short follow-up questionnaire consisting of 14-21 items regarding cessation assistance utilization, smoking cessation status, stages of decision making, and knowledge.

After 6 months (t=3) and 12 months (t=4), users are asked to fill in a longer follow-up questionnaire that consists of 16-88 items relating to cessation assistance utilization, smoking abstinence (7-day point prevalence abstinence and prolonged abstinence), and questions regarding smoking behavior for those that did not achieve smoking abstinence. Health care utilization, productivity losses, quality of life, and decisional regret are also assessed (again). During t=4, 2 qualitative items are included to assess users’ experience of having used the DA during their cessation attempt.

All included measures are based on our theoretical background (ie, SDT [47,48]), the IPDAS guidelines on establishing effectiveness of DAs [49], Dutch guidelines on health economic evaluation [50], and expert knowledge regarding smoking cessation outcomes [51,52]. For a more detailed overview of all included constructs, measurements, and their respective sources, see Table 3.

**Table 3:** Overview of included constructs, measurements, and sources.

Constructs		Measurements and sources	Purpose
**Baseline (t=0): directly before the decision aid**			
	Demographics	Age, gender, education [52]	Sample description, attrition analyses, covariate(s) in (main) analyses
	Smoking behavior	Smoking status, motivation to quit, type of tobacco products, amount of tobacco consumption, amount of past cessation attempts, and cessation assistance utilization in the past 6 months [52]	Sample description, attrition analyses
	Nicotine dependence	Revised Fagerström Test for Nicotine Dependence (FTND-R) [53]	Sample description, attrition analyses, covariate in (main) analyses
	Productivity loss	iMTA Productivity Cost Questionnaire (iPCQ) [54]	Economic evaluation
	Health care utilization	Contacts with health care professionals (plus frequency) in the past 6 months, cessation assistance utilization (plus frequency) in the past 6 months [55]	Economic evaluation
	Quality of life	ICEpop CAPability measure for Adults (ICECAP-A) [56]	Economic evaluation
	Stages of decision making	Stage of Decision Making [57]	Sample description, attrition analyses, process information, covariate in (main) analyses
**Follow-up (t=1): directly after the decision aid**			
	Stages of decision making	Stage of Decision Making [57]	Sample description, process information
	Decision	Decision after having used the decision aid (DA), not yet implemented	Sample description, process information
	Decision-making process	Decisional conflict scale [58], first item from the Preparation for Decision Making Scale [59]	Hypothesis testing, H_4_ (decisional conflict scale [58]); process information and additional studies (see “Data Analysis”)
	Knowledge	Self-developed knowledge scale [60]	Additional studies (see “Data Analysis”)
	Perceived autonomy support	The Virtual Care Climate Questionnaire [47]	Additional studies (see “Data Analysis”)
	Perceived competence	Perceived competence scale [48]	Additional studies (see “Data Analysis”)
	Evaluation questions	Regarding attention, clarity, satisfaction, and one open question	Process information
	Recruitment channels	N/A^a^	Recruitment monitoring and analyses
**Follow-up (t=2): 1 month after baseline**			
	Decision implementation	Implemented decision (choice) after having used the DA	Hypothesis testing, H_2a_ and H_3a_
	Smoking (cessation) behavior	Prolonged abstinence; 7-day point prevalence abstinence; for users that did not successfully stop: type of tobacco products and amount of tobacco consumption [52]	Hypothesis testing, H_1a_ (7-day point prevalence abstinence [52]); additional studies (see “Data Analysis”)
	Stages of decision making	Stage of Decision Making [57]	Sample description, process information
	Knowledge	Self-developed knowledge scale [60]	Additional studies (see “Data Analysis”)
**Follow-up (t=3): 6 months after baseline**			
	Decision implementation	Implemented decision (choice) after having used the DA	Hypothesis testing, H_2b_ and H_3b_
	Smoking (cessation) behavior	Prolonged abstinence; 7-day point prevalence abstinence; for users that did not successfully stop: type of tobacco products and amount of tobacco consumption [52]	Hypothesis testing, H_1b_ and H_3a_ (7-day point prevalence abstinence [52]); additional studies (see “Data Analysis”)
	Productivity loss	iPCQ [54]	Economic evaluation
	Health care utilization	Contacts with health care professionals (plus frequency) in the past 6 months, cessation assistance utilization (plus frequency) in the past 6 months [55]	Economic evaluation
	Quality of life	ICECAP-A [56]	Economic evaluation
	Decisional regret	Decision regret scale [61]	Additional studies (see “Data Analysis”)
**Follow-up (t=4): 12 months after baseline**			
	Decision implementation	Implemented decision (choice) after having used the DA	Hypothesis testing, H_2c_
	Smoking (cessation) behavior	Prolonged abstinence; 7-day point prevalence abstinence; for users that did not successfully stop: type of tobacco products and amount of tobacco consumption [52]	Hypothesis testing, H_1c_ and H_3b_ (7-day point prevalence abstinence [52]); additional studies (see “Data Analysis”)
	Productivity loss	iPCQ [54]	Economic evaluation
	Health care utilization	Contacts with health care professionals (plus frequency) in the past 6 months, cessation assistance utilization (plus frequency) in the past 6 months [55]	Economic evaluation
	Quality of life	ICECAP-A [56]	Economic evaluation
	Decisional regret	Decision regret scale [61]	Additional studies (see “Data Analysis”)
	Evaluation questions	Regarding perceived decision support	Process information

^a^N/A: not applicable.

**Figure 4:**
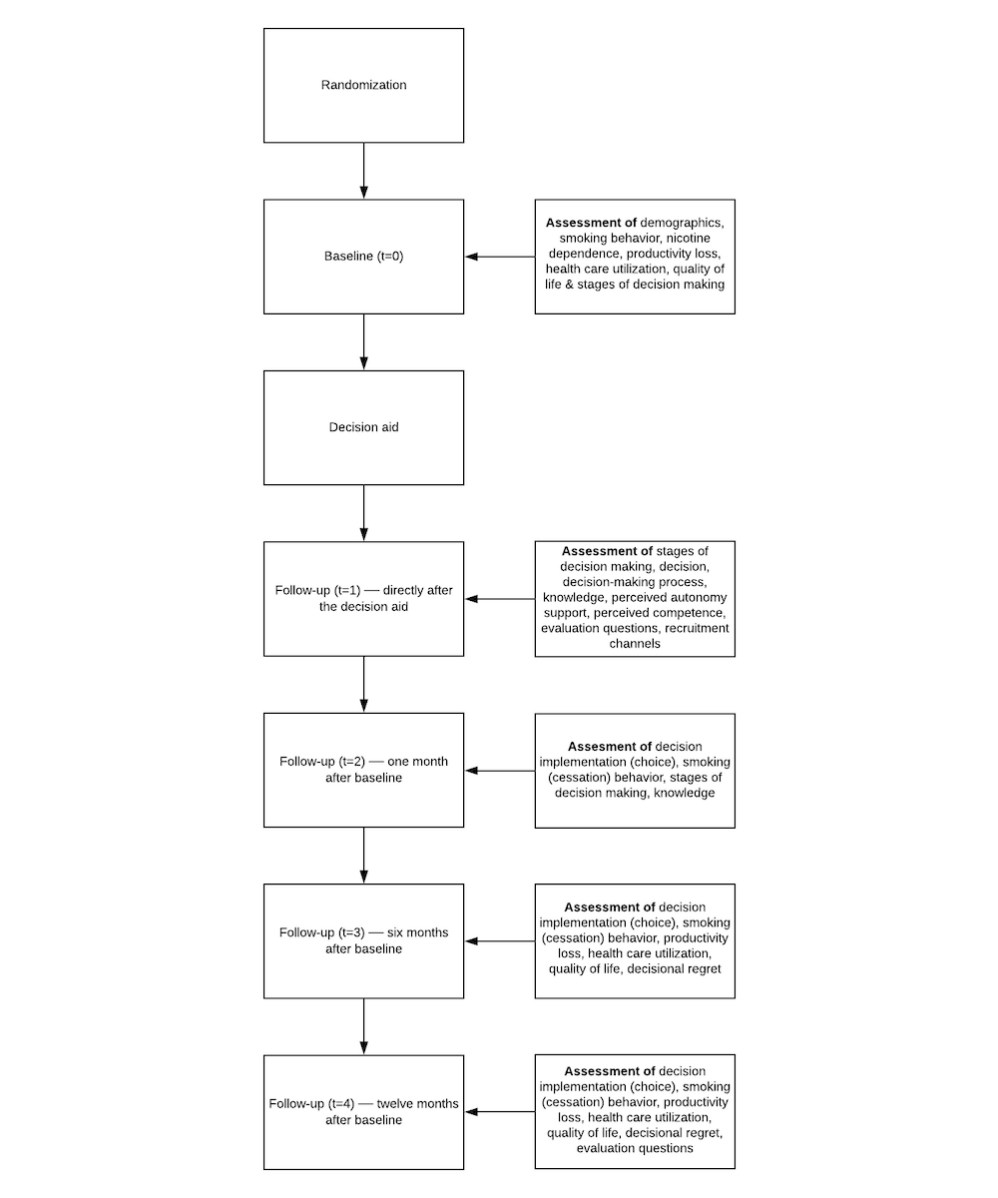
Trial flow.

Participants are invited to fill in each follow-up questionnaire if they made use of the entire DA, even when they skipped one of the other follow-up questionnaires. To avoid high drop-out rates, participants receive either 1 automatic reminder after a week (if they have not filled in the follow-up questionnaires at all) or 2 after 2 days and a week (if they already started filling in at least parts of the follow-up questionnaires). Participants that started using either the DA or started filling in the baseline questionnaire (t=0) without finishing it also receive 2 automatic reminders (after 2 days and a week). In addition, participants who took part in the last measurement receive €10 (US $11.84). A visual representation of the trial flow can be seen in Figure 4.

### Data Analysis

#### Assessment of the Scale Quality

Before conducting any analyses, we will assess the scale quality of all measurement instruments where appropriate, following the 2 steps proposed by Crutzen and Peters [62]: (1) conformation of intended structure of the measurement instrument by means of exploratory factor analyses and (2) calculation of omega [63] as a less biased alternative to (Cronbach) α. Compared to α, omega has more realistic assumptions regarding variances of and covariance between items [64]. Omega_hierarchical_ is based upon the sum of the squared loadings of items on the general factor and reduces the risks of misjudging the internal consistency of scientific scales [65]. Values will be calculated with R [66], making use of the integrated development environment RStudio [67].

#### Analysis of Primary and Secondary Outcomes

To assess the effects of the DA on dichotomous and continuous (primary and secondary) outcome measures, logistic and linear regression analyses will be conducted, respectively. Mediation analyses will be conducted to determine whether the effects of the DA on smoking abstinence are mediated through cessation assistance use. All analyses will include covariates that were selected a priori (as recommended by Gruijters [68] and De Boer et al [69]), if these are also associated with the 3 outcome measures as described in our hypotheses (ie, smoking abstinence, evidence-based cessation assistance use, and decisional conflict) within our sample. Demographic factors (ie, age, gender, and education) were selected for all 3 outcome measures. The Revised Fagerström Test for Nicotine Dependence was also selected for the smoking-related outcome measures, whereas stage of decision making was selected for decisional conflict. More information on the rationale for selecting those covariates can be found in Multimedia Appendix 1*.* Results will be reported from both the fully adjusted as well as crude analyses [68]. These analyses as well will be done with R [66], making use of the integrated development environment RStudio [67]. To test the robustness of the results, all analyses will be conducted according to 3 different approaches (if applicable): analyses based on (1) worst case scenario (dropout respondents are considered not to have changed), (2) multiple imputations, and (3) complete cases only. We will also test whether selective dropout has occurred in order to subsequently minimize the bias that this can cause.

#### Economic Evaluation

An economic evaluation will be performed from a societal perspective with a time horizon of 12 months. Both a cost-effectiveness analysis and a cost-utility analysis will be conducted. For the cost-effectiveness analysis, 2 incremental cost-effectiveness ratios will be calculated, based on the cost per abstinent respondent and the cost per individual who uses evidence-based cessation assistance. For the cost-utility analysis, an incremental cost-utility ratio will be calculated, based on the ICEpop CAPability measure for Adults, as proposed by Smit et al [70]. Uncertainty will be accounted for by bootstrapping and several univariate and multivariate sensitivity analyses. Cost-effectiveness acceptability curves will be constructed, showing for varying willingness-to-pay thresholds the probability that the DA with an explicit VCM and tailored advice is cost-effective compared to the DA without an explicit VCM and tailored advice.

### Additional Studies

An additional study using the data collected during the RCT as described in this protocol will be conducted in the future to test the cognitive processes (eg, the clarification of one’s values) that may underlie the effects of the DA on primary (ie, smoking abstinence) and secondary (eg, decisional regret) outcomes activated by the DAs by making use of Structural Equation Modelling in R [66] with the lavaan package [71].

## Results

The RCT started in January 2020, and at this writing, 1248 users created an account, of which 519 finished the DA. Data collection is ongoing and will be conducted until September 2021.

## Discussion

This paper describes the systematic development of an autonomy-supportive DA to assist smokers in choosing evidence-based cessation assistance and the intended study design to test its (cost-)effectiveness. In order to systematically develop an evidence-based online DA for a lifestyle behavior, we applied the IPDAS development process guideline [30]. The DA described in this article does not require the assistance of a health care professional and is intended for all adults that want to quit smoking in the near future (ie, within the coming 6 months). Therefore, we explicitly try to reach a broad range of potential users by focusing our recruitment strategy on social media platforms and other, more traditional media outlets. This enables us to reach a target group interested in making a decision on smoking cessation support independently from more traditional channels, such as their health care provider. This is especially interesting, as Dutch smokers often do not engage in smoking cessation discussions with their health care provider and instead most often turn to the internet for smoking cessation advice [8]; therefore, we believe this recruitment strategy is most suitable for reaching this rather general target group and could expose an additional group of smokers motivated to quit to evidence-based cessation assistance.

The results of this RCT can be used to improve our understanding of decision-making processes (especially in the context of smoking cessation) and to provide new insights into effective elements of DAs, to support not only informed decision making but also subsequent behavioral change. Formative studies (ie, the aforementioned interviews and beta/usability tests) have shown that potential end users are interested in the DA as they want to achieve long-term behavior change (ie, smoking abstinence). However, most RCTs testing the effects of DAs focus on decisional outcomes alone [9]. Our RCT will thus be of added value to the field and might provide unique insights that have remained unexplored so far.

Furthermore, if the DA will be proven to be (cost-)effective, it can be implemented nationwide and will thus help to reduce tobacco-related diseases and deaths. Two of 3 unique characteristics of this DA that were mentioned in the Introduction to overcome the limitation of the paper-based DA that was tested in the Netherlands before [14] could make a nationwide implementation particularly interesting: The online nature will (1) allow for a wider reach and (2) enable a more flexible and interactive approach.

### Potential Strengths of the Study

The study shows several potential strengths. First, we are conducting an RCT to assess the DA’s impact on behavior change and decision-making outcomes, with randomization being done automatically. The fact that participants are being blinded (as the control group receives a DA as well) further strengthens this study. DAs aimed at smoking cessation with explicit VCMs have been tested before, but without following an RCT protocol (eg, [25]). Testing the DA in a longitudinal RCT design will allow for stronger conclusions about the DA’s impact. Following participants for a longer period of time is especially interesting as explicit VCMs have been shown to result in long-term benefits but not necessarily in short-term benefits [21].

Second, recruited end users are only included if they are motivated to stop smoking in the near future, meaning they actually have to decide how they wish to stop smoking. A previous study that tested the effects of an explicit VCM found no effects [46]; however, they made use of a hypothetical decision, which hampers interpretation of their findings. As both primary (ie, smoking abstinence) and secondary (eg, decisional conflict) outcomes relate to “real-life” phenomes and affect, testing the DA in a nonhypothetical and “natural” context will also allow for stronger conclusions about the DA’s impact.

Third, and as previously mentioned, the DA is tested not only for outcomes at the decisional level but also at the behavioral level. Above and beyond this, the DA will also be tested regarding its cost-effectiveness. This is especially interesting as experts strongly urge to test eHealth applications not only for their effectiveness but for their cost-effectiveness as well [72]. However, these practices are not commonly applied. Testing the DA’s cost-effectiveness will enable decision makers to make evidence-based recommendations regarding the widespread implementation of the cessation assistance DA — which is particularly interesting given the scarcity of health care resources.

### Potential Limitations of the Study

The study also has a few potential limitations. First, as it was decided to include 2, and not 3, study arms in our RCT, it will not be possible to assess the effects of the explicit VCM and the tailored advice separately. However, including a third arm would have required an even bigger sample, which was deemed not feasible.

Second, the online nature of both the DA and our recruitment strategy exclude potential participants that either have no access to the internet or lack the digital skills needed to use the DA. However, given that the Netherlands is 1 of the 2 countries with the highest percentage of households with internet access in the European Union [73], we expect this to be a relatively minor limitation.

### Conclusion

DAs that assist smokers in choosing evidence-based cessation assistance offer a potential approach to both counteract the public health effects of smoking and facilitate individual’s smoking cessation attempts. However, knowledge regarding effective elements and (cost-)effectiveness is lacking. Our study is therefore expected to contribute significantly to the current knowledge regarding smoking cessation DA application, the added value of explicit VCMs within such DAs, and the effect on behavioral and informed decision-making outcomes.

## Appendix

Multimedia Appendix 1 Selection of covariates.

## Abbreviations

DA: decision aid
FTND-R: Revised Fagerström Test for Nicotine Dependence
ICECAP-A: ICEpop CAPability measure for Adults
iPCQ: iMTA Productivity Cost Questionnaire
IPDAS: International Patient Decision Aid Standards
NRT: nicotine replacement therapy
RCT: randomized controlled trial
SDT: Self-Determination Theory
VCM: value clarification method

## References

[1] (2014). The Health Consequences of Smoking–50 Years of Progress: A Report of the Surgeon General. U.S. Department of Health and Human Services.

[2] Springvloet L, Bommelé J, Willemsen M, van Laar M (2018). Kerncijfers roken 2017. Trimbos-instituut.

[3] (2009). WHO report on the global tobacco epidemic 2009: Implementing smoke-free environments. World Health Organization.

[4] Cahill K, Stevens S, Perera R, Lancaster T (2013). Pharmacological interventions for smoking cessation: an overview and network meta-analysis. Cochrane Database Syst Rev.

[5] Lancaster T, Stead LF (2017). Individual behavioural counselling for smoking cessation. Cochrane Database Syst Rev.

[6] White AR, Rampes H, Liu JP, Stead LF, Campbell J (2011). Acupuncture and related interventions for smoking cessation. Cochrane Database Syst Rev.

[7] Zhu S, Melcer T, Sun J, Rosbrook B, Pierce JP (2000). Smoking cessation with and without assistance: a population-based analysis. Am J Prev Med.

[8] Borland R, Li L, Driezen P, Wilson N, Hammond D, Thompson ME, Fong GT, Mons U, Willemsen MC, McNeill A, Thrasher JF, Cummings KM (2012). Cessation assistance reported by smokers in 15 countries participating in the International Tobacco Control (ITC) policy evaluation surveys. Addiction.

[9] Stacey D, Légaré F, Lewis K, Barry MJ, Bennett CL, Eden KB, Holmes-Rovner M, Llewellyn-Thomas H, Lyddiatt A, Thomson R, Trevena L (2017). Decision aids for people facing health treatment or screening decisions. Cochrane Database Syst Rev.

[10] Montori VM, LeBlanc A, Buchholz A, Stilwell DL, Tsapas A (2013). Basing information on comprehensive, critically appraised, and up-to-date syntheses of the scientific evidence: a quality dimension of the International Patient Decision Aid Standards. BMC Med Inform Decis Mak.

[11] Moyo F, Archibald E, Slyer JT (2018). Effectiveness of decision aids for smoking cessation in adults: a quantitative systematic review. JBI Database System Rev Implement Rep.

[12] Su YL, Reeve J (2011). A Meta-analysis of the Effectiveness of Intervention Programs Designed to Support Autonomy. Educ Psychol Rev.

[13] Ryan RM, Patrick H, Deci EL, Williams GC (2008). Facilitating health behaviour change and its maintenance: Interventions based on self-determination theory. European Health Psychologist.

[14] Willemsen MC, Wiebing M, van Emst A, Zeeman G (2006). Helping smokers to decide on the use of efficacious smoking cessation methods: a randomized controlled trial of a decision aid. Addiction.

[15] Cheung KL, Wijnen B, de Vries H (2017). A Review of the Theoretical Basis, Effects, and Cost Effectiveness of Online Smoking Cessation Interventions in the Netherlands: A Mixed-Methods Approach. J Med Internet Res.

[16] Van de Belt TH, Engelen LJLPG, Berben SAA, Teerenstra S, Samsom M, Schoonhoven L (2013). Internet and social media for health-related information and communication in health care: preferences of the Dutch general population. J Med Internet Res.

[17] Bol N, Høie NM, Nguyen MH, Smit ES (2019). Customization in mobile health apps: explaining effects on physical activity intentions by the need for autonomy. Digit Health.

[18] Brazier JE, Dixon S, Ratcliffe J (2009). The role of patient preferences in cost-effectiveness analysis: a conflict of values?. Pharmacoeconomics.

[19] Brewin CR, Bradley C (1989). Patient preferences and randomised clinical trials. BMJ.

[20] Fagerlin A, Pignone M, Abhyankar P, Col N, Feldman-Stewart D, Gavaruzzi T, Kryworuchko J, Levin CA, Pieterse AH, Reyna V, Stiggelbout A, Scherer LD, Wills C, Witteman HO (2013). Clarifying values: an updated review. BMC Med Inform Decis Mak.

[21] Feldman-Stewart D, Tong C, Siemens R, Alibhai S, Pickles T, Robinson J, Brundage MD (2012). The impact of explicit values clarification exercises in a patient decision aid emerges after the decision is actually made: evidence from a randomized controlled trial. Med Decis Making.

[22] Abhyankar P, Bekker HL, Summers BA, Velikova G (2011). Why values elicitation techniques enable people to make informed decisions about cancer trial participation. Health Expect.

[23] Witteman HO, Julien AS, Ndjaboue R, Exe NL, Kahn VC, Fagerlin A, Zikmund-Fisher BJ (2020). What Helps People Make Values-Congruent Medical Decisions? Eleven Strategies Tested across 6 Studies. Med Decis Making.

[24] Witteman HO, Gavaruzzi T, Scherer LD, Pieterse AH, Fuhrel-Forbis A, Chipenda Dansokho S, Exe N, Kahn VC, Feldman-Stewart D, Col NF, Turgeon AF, Fagerlin A (2016). Effects of Design Features of Explicit Values Clarification Methods: A Systematic Review. Med Decis Making.

[25] Cupertino AP, Richter K, Cox LS, Garrett S, Ramirez R, Mujica F, Ellerbeck EF (2010). Feasibility of a Spanish/English computerized decision aid to facilitate smoking cessation efforts in underserved communities. J Health Care Poor Underserved.

[26] Eysenbach G, CONSORT-EHEALTH Group (2011). CONSORT-EHEALTH: improving and standardizing evaluation reports of Web-based and mobile health interventions. J Med Internet Res.

[27] Peters GJY, Abraham C, Crutzen R (2012). Full disclosure: doing behavioural science necessitates sharing. European Health Psychologist.

[28] Keuzehulp Visor (@keuzehulp_visor).

[29] Smith A, Anderson M (2018). Social Media Use in 2018. Pew Research Center.

[30] Coulter A, Stilwell D, Kryworuchko J, Mullen PD, Ng CJ, van der Weijden T (2013). A systematic development process for patient decision aids. BMC Med Inform Decis Mak.

[31] Feldman-Stewart D, O'Brien MA, Clayman ML, Davison BJ, Jimbo M, Labrecque M, Martin RW, Shepherd H (2013). Providing information about options in patient decision aids. BMC Med Inform Decis Mak.

[32] Jaspers MWM (2009). A comparison of usability methods for testing interactive health technologies: methodological aspects and empirical evidence. Int J Med Inform.

[33] Altendorf MB, van Weert JCM, Hoving C, Smit ES (2019). Should or could? Testing the use of autonomy-supportive language and the provision of choice in online computer-tailored alcohol reduction communication. Digit Health.

[34] Maddux JE, Rogers RW (1983). Protection motivation and self-efficacy: A revised theory of fear appeals and attitude change. Journal of Experimental Social Psychology.

[35] OverNite Software Europe (2020). Create online questionnaires with personal feedback. TailorBuilder.

[36] de Vries M, Fagerlin A, Witteman HO, Scherer LD (2013). Combining deliberation and intuition in patient decision support. Patient Educ Couns.

[37] Durand MA, Witt J, Joseph-Williams N, Newcombe RG, Politi MC, Sivell S, Elwyn G (2015). Minimum standards for the certification of patient decision support interventions: feasibility and application. Patient Educ Couns.

[38] Abhyankar P, Volk RJ, Blumenthal-Barby J, Bravo P, Buchholz A, Ozanne E, Vidal DC, Col N, Stalmeier P (2013). Balancing the presentation of information and options in patient decision aids: an updated review. BMC Med Inform Decis Mak.

[39] Chavannes N, Drenthen T, Wind L, Van Avendonk M, Van den Donk M, Verduijn M (2017). NHG-Behandelrichtlijn: Stoppen met roken.

[40] Stead LF, Perera R, Bullen C, Mant D, Hartmann-Boyce J, Cahill K, Lancaster T (2012). Nicotine replacement therapy for smoking cessation. Cochrane Database Syst Rev.

[41] (2020). National Health Care Institute (Zorginstituut Nederland).

[42] (2020). Medicines Evaluation Board (College ter Beoordeling van Geneesmiddelen).

[43] Chernev A, Böckenholt U, Goodman J (2015). Choice overload: A conceptual review and meta-analysis. Journal of Consumer Psychology.

[44] Witteman HO, Scherer LD, Gavaruzzi T, Pieterse AH, Fuhrel-Forbis A, Chipenda Dansokho S, Exe N, Kahn VC, Feldman-Stewart D, Col NF, Turgeon AF, Fagerlin A (2016). Design Features of Explicit Values Clarification Methods: A Systematic Review. Med Decis Making.

[45] Klaassen LA, Dirksen CD, Boersma LJ, Hoving C, B-beslist!-group (2018). A novel patient decision aid for aftercare in breast cancer patients: A promising tool to reduce costs by individualizing aftercare. Breast.

[46] Sheridan SL, Griffith JM, Behrend L, Gizlice Z, Cai J, Pignone MP (2010). Effect of adding a values clarification exercise to a decision aid on heart disease prevention: a randomized trial. Med Decis Making.

[47] Smit ES, Dima AL, Immerzeel SAM, van den Putte B, Williams GC (2017). The Virtual Care Climate Questionnaire: Development and Validation of a Questionnaire Measuring Perceived Support for Autonomy in a Virtual Care Setting. J Med Internet Res.

[48] Williams GC, Freedman ZR, Deci EL (1998). Supporting autonomy to motivate patients with diabetes for glucose control. Diabetes Care.

[49] Sepucha KR, Borkhoff CM, Lally J, Levin CA, Matlock DD, Ng CJ, Ropka ME, Stacey D, Joseph-Williams N, Wills CE, Thomson R (2013). Establishing the effectiveness of patient decision aids: key constructs and measurement instruments. BMC Med Inform Decis Mak.

[50] (2016). Guideline for economic evaluations in healthcare. Zorginstituut Nederland.

[51] Cheung KL, de Ruijter D, Hiligsmann M, Elfeddali I, Hoving C, Evers SMAA, de Vries H (2017). Exploring consensus on how to measure smoking cessation. A Delphi study. BMC Public Health.

[52] Mudde AN, Willemsen MC, Kremers SPJ, de Vries H (2000). Meetinstrumenten voor onderzoek naar roken en stoppen met roken.

[53] Korte KJ, Capron DW, Zvolensky M, Schmidt NB (2013). The Fagerström test for nicotine dependence: do revisions in the item scoring enhance the psychometric properties?. Addict Behav.

[54] Bouwmans C, Krol M, Severens H, Koopmanschap M, Brouwer W, Hakkaart-van Roijen L (2015). The iMTA Productivity Cost Questionnaire: A Standardized Instrument for Measuring and Valuing Health-Related Productivity Losses. Value Health.

[55] de Ruijter D, Hoving C, Evers S, Hudales R, de Vries H, Smit E (2019). An economic evaluation of a computer-tailored e-learning program to promote smoking cessation counseling guideline adherence among practice nurses. Patient Educ Couns.

[56] Al-Janabi H, Flynn TN, Coast J (2012). Development of a self-report measure of capability wellbeing for adults: the ICECAP-A. Qual Life Res.

[57] O'Connor AM (2000). User Manual – Stage of Decision Making. Ottawa Hospital Research Institute.

[58] O'Connor AM (1995). Validation of a decisional conflict scale. Med Decis Making.

[59] Bennett C, Graham ID, Kristjansson E, Kearing SA, Clay KF, O'Connor AM (2010). Validation of a preparation for decision making scale. Patient Educ Couns.

[60] O'Connor AM (2000). User Manual – Knowledge. Ottawa Hospital Research Institute.

[61] Brehaut JC, O'Connor AM, Wood TJ, Hack TF, Siminoff L, Gordon E, Feldman-Stewart D (2003). Validation of a decision regret scale. Med Decis Making.

[62] Crutzen R, Peters GJY (2017). Scale quality: alpha is an inadequate estimate and factor-analytic evidence is needed first of all. Health Psychol Rev.

[63] McDonald RP (2013). Test theory: A unified treatment.

[64] Peters GJY (2014). The alpha and the omega of scale reliability and validity: why and how to abandon Cronbach's alpha and the route towards more comprehensive assessment of scale quality. European Health Psychologist.

[65] Dunn TJ, Baguley T, Brunsden V (2014). From alpha to omega: a practical solution to the pervasive problem of internal consistency estimation. Br J Psychol.

[66] R Core Team (2020). R: A language and environment for statistical computing.

[67] RStudio Team (2020). RStudio: Integrated Development for R.

[68] Gruijters SLK (2016). Baseline comparisons and covariate fishing: Bad statistical habits we should have broken yesterday. European Health Psychologist.

[69] de Boer MR, Waterlander WE, Kuijper LDJ, Steenhuis IHM, Twisk JWR (2015). Testing for baseline differences in randomized controlled trials: an unhealthy research behavior that is hard to eradicate. Int J Behav Nutr Phys Act.

[70] Smit ES, de Vries H, Oberjé EJM, Evers SMAA (2015). Easier Said Than Done: Overcoming Challenges in the Economic Evaluation of Internet-Based Lifestyle Interventions. European Health Psychologist.

[71] Rosseel Y (2012). lavaan: An R Package for Structural Equation Modeling. J. Stat. Soft.

[72] Ahern DK, Kreslake JM, Phalen JM (2006). What is eHealth (6): perspectives on the evolution of eHealth research. J Med Internet Res.

[73] (2018). Internet access and use statistics - households and individuals Internet. Eurostat.

