# Supplementary material for: An Autonomy-Supportive Online Decision Aid to Assist Smokers in Choosing Evidence-Based Cessation Assistance: Development Process and Protocol of a Randomized Controlled Trial
Source: JMIR Res Protoc. 2020 Dec 15;9(12):e21772. doi: 10.2196/21772 (PMC7772073; doi:10.2196/21772)
Supplement: Multimedia Appendix 1 [file resprot_v9i12e21772_app1.docx]

Appendix 1: Selection of covariates.

All analyses will include covariates that were selected a priori (as recommended by Gruijters [1] and De Boer et al. [2]), if these are also associated with the 3 outcome measures as described in our hypotheses (ie, smoking abstinence, evidence-based cessation assistance use, and decisional conflict) *within our sample*. Demographic factors (ie, age, gender, and education) were selected for all 3 outcome measures. The Revised Fagerström Test for Nicotine Dependence was also selected for the smoking-related outcome measures, whereas stages of decision making was selected for decisional conflict.

| **Outcome of interest** | **Covariates selected** | **Rationale** |
| --- | --- | --- |
| *Smoking abstinence* | Revised Fagerström Test for Nicotine Dependence (FTND-R) and demographic factors (age, gender and education) | Nicotine dependence is the only consistent predictor of successful smoking cessation in adult general populations as shown in a systematic review [3] which was also found in a review about clinical populations [4]. And while demographic factors on the other hand have not been shown to play a consistent role in smoking cessation success [3,4] — especially not in general populations [3], other reviews [4] do report that certain demographic factors (eg, age) can influence successful smoking cessation. |
| *Evidence-based cessation assistance use* | Revised Fagerström Test for Nicotine Dependence (FTND-R) and demographic factors (age, gender and education) | Previous research [5–7] has shown that both “heavy smokers” and certain demographic groups are more likely to use cessation assistance (eg, women are seemingly more likely to use cessation assistance [5]), Zhu et al. [5] also showed that smoking behavior can influence cessation assistance uptake —however, as the FTND-R already captures smoking behavior [8], it was decided to only include the FTND-R. |
| *Decisional conflict* | Stages of decision making and demographic factors (age, gender and education) | Predictors of decisional conflict are not commonly studied as far as we are aware, however, it has been shown that someone’s stage of decision making is associated with decisional conflict [9], also one’s ability to understand health information also appears to be related to decisional conflict [10], which in turn is often shown to be associated with demographic factors [11,12]. |

# References

1. Gruijters SLK. Baseline comparisons and covariate fishing: Bad statistical habits we should have broken yesterday. Eur Health Psychol 2016;18(5):205–209.

2. De Boer MR, Waterlander WE, Kuijper LDJ, Steenhuis IHM, Twisk JWR. Testing for baseline differences in randomized controlled trials: an unhealthy research behavior that is hard to eradicate. Int J Behav Nutr Phys Act Springer; 2015;12(1):4. [doi: 10.1186/s12966-015-0162-z]

3. Vangeli E, Stapleton J, Smit ES, Borland R, West R. Predictors of attempts to stop smoking and their success in adult general population samples: a systematic review. Addiction Wiley Online Library; 2011;106(12):2110–2121. [doi: 10.1111/j.1360-0443.2011.03565.x]

4. Caponnetto P, Polosa R. Common predictors of smoking cessation in clinical practice. Respir Med Elsevier; 2008;102(8):1182–1192. [doi: 10.1016/j.rmed.2008.02.017]

5. Zhu SH, Melcer T, Sun J, Rosbrook B, Pierce JP. Smoking cessation with and without assistance: A population-based analysis. Am J Prev Med 2000 May 1;18(4):305–311. [doi: 10.1016/S0749-3797(00)00124-0]

6. Smith AL, Chapman S, Dunlop SM. What do we know about unassisted smoking cessation in Australia? A systematic review, 2005–2012. Tob Control BMJ Publishing Group Ltd; 2015;24(1):18–27. [doi: 10.1136/tobaccocontrol-2013-051019]

7. Filippidis FT, Gerovasili V, Vardavas CI, Agaku IT, Tountas Y. Determinants of use of smoking cessation aids in 27 European countries. Prev Med Elsevier; 2014;65:99–102. [doi: 10.1016/j.ypmed.2014.05.004]

8. Korte KJ, Capron DW, Zvolensky M, Schmidt NB. The Fagerström test for nicotine dependence: do revisions in the item scoring enhance the psychometric properties? Addict Behav 2013;38(3):1757–1763. [doi: 10.1016/j.addbeh.2012.10.013]

9. O’Connor AM. Stage of Decision Making [document on the Internet]. Ottawa: Ottawa Hospital Research Institute; 2000 [cited 2020 Mar 24]. Available from: https://decisionaid.ohri.ca/docs/develop/User_Manuals/UM_Stage_Decision_Making.pdf

10. Pecanac KE, Brown RL, Kremsreiter HB. Decisional Conflict During Major Medical Treatment Decision-making: a Survey Study. J Gen Intern Med Springer; 2020;1–7. [doi: 10.1007/s11606-020-06125-1]

11. Davis SN, Wischhusen JW, Sutton SK, Christy SM, Chavarria EA, Sutter ME, Roy S, Meade CD, Gwede CK. Demographic and psychosocial factors associated with limited health literacy in a community-based sample of older Black Americans. Patient Educ Couns Elsevier; 2020;103(2):385–391. [doi: 10.1016/j.pec.2019.08.026]

12. Oliffe JL, McCreary DR, Black N, Flannigan R, Goldenberg SL. Canadian men’s health literacy: a nationally representative study. Health Promot Pract SAGE Publications Sage CA: Los Angeles, CA; 2019;1524839919837625. [doi: 10.1177/1524839919837625]
